# Promising therapeutic efficacy of nitazoxanide-loaded zinc oxide nano-formula against intestinal and muscular phases of experimental trichinellosis

**DOI:** 10.1371/journal.pntd.0013239

**Published:** 2025-07-18

**Authors:** Nancy Abd-elkader Hagras, Fatma Hegab, Shimaa Atta, Reham A. Gadallah, Youssef Elsayed, Gehan A. M. Khodear

**Affiliations:** 1 Medical School Program and Pharmacy Program, Alexandria National University, Alexandria, Egypt; 2 Alexandria University Hospitals, Alexandria University, Alexandria, Egypt; 3 Department of Pathology, Theodor Bilharz Research Institute, Giza, Egypt; 4 Department of Immunology, Theodor Bilharz Research Institute, Giza, Egypt; 5 Department of Clinical Chemistry, Theodor Bilharz Research Institute, Giza, Egypt; 6 Hochschule Anhalt University of Applied Sciences, Köthen, Germany; 7 Medical Technology Center, Medical Research Institute, Alexandria University, Alexandria, Egypt; NIAID-ICER, INDIA

## Abstract

Trichinellosis is a ubiquitous parasitic infection caused by a zoonotic nematode known as *Trichinella spiralis* (*T. spiralis*). It starts with the adult worm in the intestinal phase and ends up with the larva reaching the muscles. The disease generally manifests with acute gastroenteritis; however, it may regrettably lead to life-threatening myositis, myocarditis and seizures. The commercially existing chemotherapeutic regimens have numerous drawbacks including severe adverse effects, high resistance rate, poor bioavailability and low efficiency towards the muscular stage. Consequently, the current study targeted the evaluation of nitazoxanide-loaded zinc oxide nanoparticles (NTZ-loaded ZnO NPs) used for the first time in the treatment of both the intestinal and muscular phases of trichinellosis in mice. Swiss Albino mice were orally infected by 250 *T. spiralis* larvae. The experimental animals were treated with the gold standard albendazole, NTZ, ZnO NPs as well as NTZ-loaded ZnO NPs. Parasitological, biochemical (creatine kinase, alanine transaminase, aspartate transaminase, alkaline phosphatase, malondialdehyde and nitric oxide), immunological (interleukins 2 and 4) and histopathological assessments were conducted. The parasitological results denoted that the mice treated with NTZ-loaded ZnO NPs revealed the uppermost significant drug efficacy (>97%) in both the intestinal and muscular phases indicating efficacious tissue penetration. Additionally, this group revealed the most profound amelioration of the biochemical and immunological markers as well as restoration of the histopathological picture. Conclusively, the present work implied a bird’s eye view on the promising effectiveness of NTZ-loaded ZnO NPs in the treatment of murine trichinellosis relying on the anti-parasitic safe nature of the formulation.

## Author summary

Trichinellosis is a disease that arises from the infection with a food-borne parasite known as *Trichinella spiralis*. The parasite presents a global distribution. Infection is acquired by consuming infected undercooked meat, mostly pork. Throughout the initial infection phase, worms invade the intestine resulting into abdominal pain, diarrhea and vomiting. Almost a week after infection, larvae start migration to the muscles, leading to muscle pain and weakness. Symptoms may aggressively complicate to heart failure, respiratory failure or central nervous system involvement. Those severe complications may unfortunately be fatal. Although there are multiple treatment options, they still have objectionable drawbacks. The commercially available drugs are not effective in eradicating the parasite muscular stage, in addition to being unsafe for pregnant women and children below three years. Nitazoxanide is a safe therapeutic option but has a lower comparable efficiency. Accordingly, this study focused on attaining an improved treatment outcome via the preparation of nano-sized formulation. The formula was attained by preparing the safe anti-microbial zinc oxide nanoparticles then loading them with nitazoxanide. The nano-formulation exhibited an outstanding propitious effectiveness against experimental trichinellosis.

## Introduction

*Trichinella spiralis* (*T. spiralis*) is a zoonotic nematode that infects a wide variety of vertebrate hosts including human [1,2]. It displays an obvious global abundance with an annual infection burden of about 10,000 cases and a mortality rate of 0.2%. In Egypt, human infection has been documented [3]. Human gets the infection through the ingestion of undercooked meat specially pork that carries the encysted *T. spiralis* larvae in the animal muscular tissues [4]. The parasite owes a unique intracellular life cycle that alternates between enteral and parenteral stages in the same host [4]. The cycle starts with the development of the adult worm in the host’s small intestine throughout the first week of infection triggering a severe pathological villi injury along with acute inflammatory infiltrates. This intestinal phase is clinically manifested by fever, nausea, vomiting, abdominal pain and diarrhea. The cycle ends up with the parenteral phase where the newborn larvae migrate through the circulation till reaching the host’s muscles. Herein, the monocytes transform into nurse cells to preserve the encapsulated larvae’s life. At this phase, the patient severely suffers from myalgia and serious muscle weakness which eventually may be complicated by respiratory failure and myocarditis. Encephalitis may also occur as a result of the larval penetration to the CNS. All those forementioned complications may unfortunately end up with death [2,4,5].

There are numerous treatments used for trichinellosis [3,4,6,7]. Albendazole and mebendazole are approved therapeutic options, however they exhibit a remarkable low effectiveness on the encapsulated larval stage. Additionally, they present a low bioavailability, high resistance degree as well as bone marrow suppression. Unfortunately, their use is also prohibited in children below three years and in pregnant females [3,4,7]. Ivermectin can also be used in the treatment of trichinellosis, yet further resistance is still probable [6]. Consequently, there is a decisive need to replace the currently available treatment options with a safer and highly effective drug against both the intestinal and muscular stages [1].

Nitazoxanide (NTZ) was primarily described as a broad-spectrum anti-protozoal, anthelmintic and anti-viral treatment [1,8]. NTZ is officially indicated for the treatment of giardiasis and cryptosporidiosis. The efficiency of NTZ has been highlighted against several helminths as well, including *Hymenolepis nana*, *Ascaris lumbricoides* and *Trichuris trichiura* [1,9,10]. It presented also a remarkable protoscolicidal efficacy against hydatid cysts [11]. Lately, NTZ has proved its therapeutic efficacy against both enteral and parenteral phases of trichinellosis [1]. NTZ is renowned with its immunomodulatory mechanism of action regarding Th1 and Th2 cytokines along with its extensive tolerance level without mutagenic or teratogenic consequences. This emphasizes its suitability for use in children and pregnant women [12,13]. All those privileges advocate its use to conquer the intestinal and muscular stages of trichinellosis. Nevertheless, it possesses a low solubility level that may hamper its efficacy. Thus, there is an urgency for the development of NTZ carrier so as to benefit from its spectacular advantages [1,14].

Nanotechnology has lately presented a new frontier in curing numerous diseases as it owes the potent ability in enhancing the drug formula. Nanoparticles (NPs) guarantee the successful transport of drugs across the entire body tissues via enhancing drug solubility besides improving tissue penetrability [15–18].

Metal oxide NPs are gaining a noteworthy attention for their anti-bacterial, anti-fungal, anti-viral, anti-protozoal and anthelmintic effects. Zinc oxide (ZnO) NPs have obtained a phenomenal interest because of their biocompatibility and safety for human use which have been officially accepted by the Food and Drug Administration (FDA) [19–21]. ZnO NPs present a remarkable cost-effective strategy owing to their anti-microbial action at low concentrations as well as their ability to combat the multi-drug resistance. ZnO mechanism of action relies on the particles size that lays in the nano-range, thus succeeding in covering a large surface area of the microbial surface, binding to it electrostatically, damaging it, ending up with microbial cell leakage and death. Subsequently, loading an anti-microbial drug on ZnO NPs can synergistically boost the antimicrobial activity. ZnO NPs serve additionally as a powerful next-generation drug delivery agent through aiding in enhancing the loaded drug stability, solubility and biodistribution [19–22].

Hence, the current work aimed to assess the effectiveness of NTZ-loaded ZnO NPs for the first time in the treatment of both the intestinal and muscular phases of murine trichinellosis compared to the albendazole (reference drug), NTZ and blank ZnO NPs. Parasitological, biochemical, immunological and histopathological parameters were undertaken.

## Results

### 1. Characterization of both blank ZnO NPs and NTZ-loaded ZnO NPs

#### 1.1. Transmission electron microscopy (TEM).

**Fig 1:**
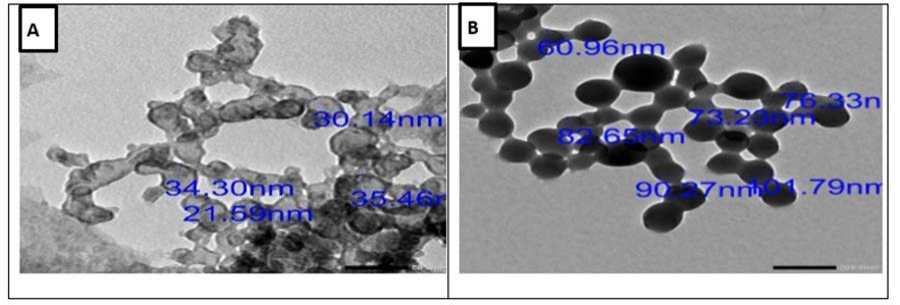
TEM micrographs of prepared NPs showing spherical and hexagonal shapes with weak agglomeration and increasing the size after NTZ loading. (A) Blank ZnO NPs (scale bar of 50 nm). (B) NTZ-loaded ZnO NPs (scale bar of 200 nm).

TEM analysis of the prepared NPs showed uniform spherical and hexagonal shapes with weak agglomeration. The sizes of the blank ZnO NPs and NTZ-loaded ZnO NPs were 30.37 ± 2.37 and 80.87 ± 5.56 nm respectively (Fig 1).

#### 1.2. Fourier transform infrared (FTIR) spectroscopy.

**Fig 2:**
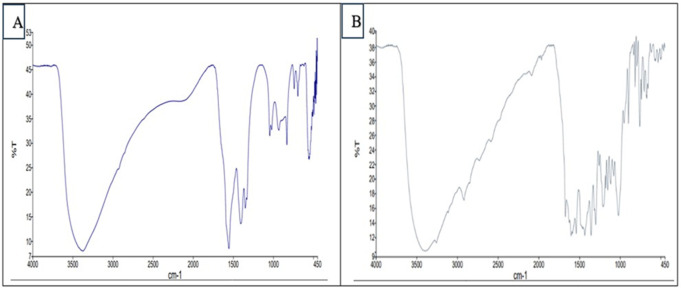
FTIR micrographs of the prepared NPs. (A) Blank ZnO NPs. (B) NTZ-loaded ZnO NPs.

FTIR was employed to verify the production of NPs through the analysis of the chemical changes in the prepared formulae. The absorption peaks of the formed blank ZnO NPs and NTZ-loaded ZnO NPs indicated the characteristic functional groups. The peaks (598.88 and 609.88 cm^-1^) that appeared in the blank ZnO NPs and the peaks (464.04, 501.74, 533.39 and 563.83 cm^-1^) that appeared in NTZ-loaded ZnO NPs represented metal-oxygen (ZnO stretching vibrations). Strong C=C bending appeared at 673.47 and 704.94 cm^-1^ for blank ZnO NPs while it appeared at 616.72, 671.27, 704.94, 738.07, 758.45, 793.75, 815.13 and 832.3 cm^-1^ for NTZ-loaded ZnO NPs. Strong C-H bending was denoted at 900.15 cm^-1^ in blank ZnO NPs and 897.76 cm^-1^ in NTZ-loaded ZnO NPs. Blank ZnO peaks (1015.98, 1032.81 and 1050.57 cm^-1^) and NTZ-loaded ZnO NPs peaks (1020.31 and 1078.83 cm^-1^) were attributed to the stretching vibration of C-N bond of the primary amine or to that of the C=O bond of primary alcohol. The peak at (1109.09, 1315.32, 1345.92, 1407.52 and 1436.07 cm^-1^) for blank ZnO while the peaks (1115.44, 1152.18, 1176.33, 1209.42, 1258.08, 1300.88, 1357.05, 1434.84 and 1462.86 cm^-1^) for NTZ-loaded ZnO NPs were ascribed to the primary and secondary alcohol in-plane bend or vibration. In NTZ-loaded ZnO NPs, peaks at 1540,12 and 1587.19 cm^-1^ corresponded to strong the N=O stretching (nitro group) that is present in NTZ drug. Blank ZnO NPs peak at 1659.82 cm^-1^ and NTZ-loaded ZnO NPs peaks at 1603.08, 1626.62 and 1673.52 cm^-1^ denoted the vibration modes of aromatic nitro compounds and alkyl that might be due to the acetate group (the precursor used for the synthesis of ZnO NPs). In blank ZnO NPs, peaks at 1906 and 2052 cm^-1^ represented medium C=C=C stretching (allene). Peaks of blank ZnO NPs at 2828.29, 2920.18 and 2942.08 cm^-1^ while peaks of NTZ-loaded ZnO NPs at 2919.05 and 3259.92 cm^-1^ signified the C–H stretching (alkanes). The overall FTIR results demonstrated the successful formation of both blank ZnO NPs and NTZ-loaded ZnO NPs (Fig 2).

#### 1.3. X-ray diffractometry (XRD).

**Fig 3:**
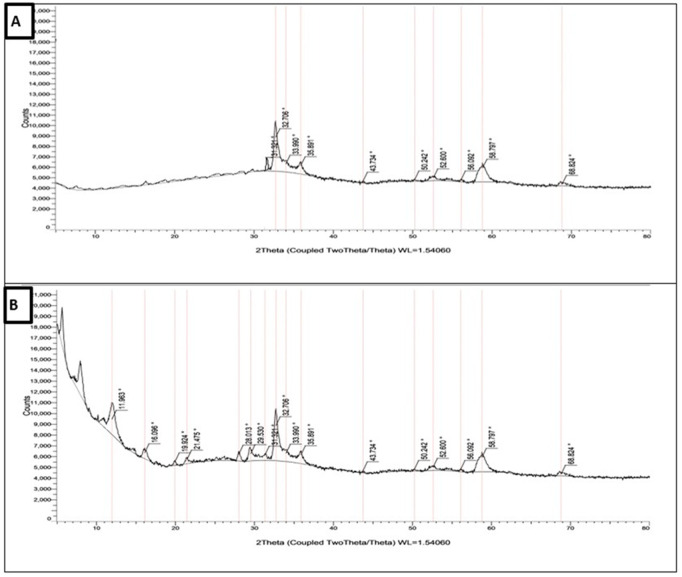
X.ray diffraction pattern of the prepared NPs. (A) Blank ZnO NPs. (B) NTZ-loaded ZnO NPs.

The crystalline nature of the prepared NPs was determined by XRD analysis. The main distinct observed diffraction peaks of the prepared blank ZnO NPs at 2θ values were (31.322 °, 32.706 °, 33.990 °, 35.891 °, 43.734 °, 50.242 °, 52.600 °, 56.092 °, 58.797 ° and 68.824 °). On the other hand, the diffraction peaks of NTZ-loaded ZnO NPs were (11.963 °, 16.096 °, 19.924°, 21.475 °, 28.013 °, 29.530 °, 31.322 °, 32.706 °, 33.990 °, 35.891 °, 43.734 °, 50.242 °, 52.600 °, 56.092 °, 58.797 ° and 68.824 °). The sharp diffraction peaks of the prepared NPs indicated their good crystallinity. The XRD pattern of the prepared NPs corresponded to spherical and hexagonal crystal structures (Fig 3).

#### 1.4. Entrapment efﬁciency (EE %).

The EE % of NTZ within ZnO NPs represented 66% relying on UV spectroscopy at 340 nm.

### 2. Evaluation of the therapeutic efficacy

#### 2.1. Parasitological assessment (*T. spiralis* adults and larvae counts and drug efficacy).

**Fig 4:**
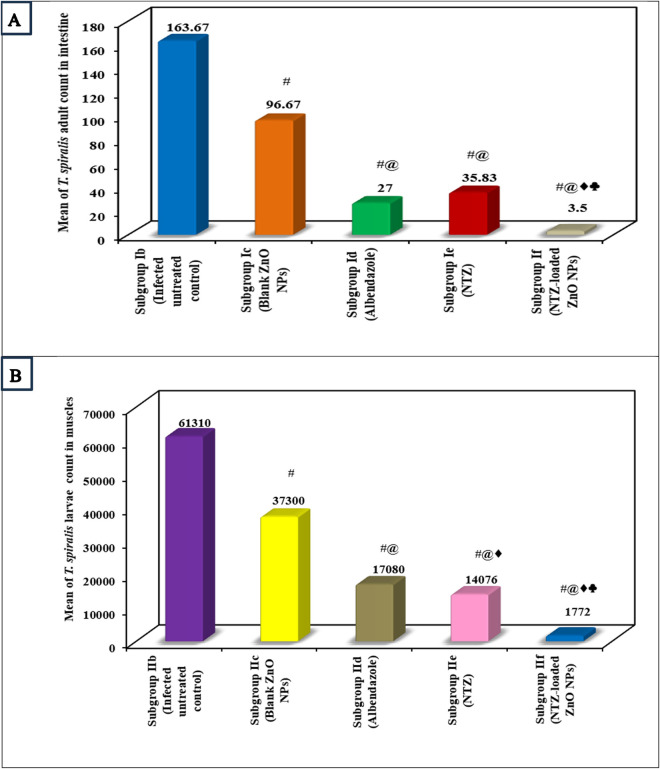
Counts of *T. spiralis.* (A) Mean count of *T. spiralis* adult worms in the intestine. (B) Mean count of *T. spiralis* larvae in muscles.

The adults and larval counts of the infected untreated control revealed the highest mean of 163.67 and 61310 respectively. All the treated subgroups presented a significant reduction in the adults and larvae counts compared to infected untreated mice. Nevertheless, NTZ-loaded ZnO NPs treated mice exhibited the highest remarkable drug efficacy (i.e., lowest mean adults and larval counts) which exceeded 97% (Fig 4 and Tables 1 and 2).

**Table 1:** Counts of adult *T. spiralis* in the intestine and drug efficacy in the various subgroups.

	Subgroup I b (Infected untreated control) (n = 6)	Subgroup I c (Blank ZnO NPs) (n = 6)	Subgroup I d (Albendazole) (n = 6)	Subgroup I e (NTZ) (n = 6)	Subgroup I f (NTZ-loaded ZnO NPs) (n = 6)
***T. spiralis* adults count in intestine**					
Min.	150	85	22	30	0
Max.	180	110	32	42	7
Mean	163.67	96.67^#^	27.0^#@^	35.83^#@^	3.50^#@♦♣^
±SD.	13.95	8.38	3.90	4.45	2.88
** F (p)**	409.173^*^ (<0.001^*^)				
**p**_**1**_		<0.001^*^	<0.001^*^	<0.001^*^	<0.001^*^
**p**_**2**_			<0.001^*^	<0.001^*^	<0.001^*^
**Sig. bet. grps.**			p_3_ = 0.319, p_4_ < 0.001^*^, p_5_ < 0.001^*^		
**Drug efficacy**		**40.94**	**83.50**	**78.11**	**97.86**

SD: **Standard deviation**

**Drug efficacy**: according to infected untreated control

**F: F for One way ANOVA test. Post Hoc Test (Tukey)** was done for pairwise comparison amongst each 2 subgroups

p: p value for comparison amongst the different subgroups

p_1_: p value for comparison amongst **Subgroup I b** and each other subgroup

p_2_: p value for comparison amongst **Subgroup I c** and each other subgroup

p_3_: p value for comparison amongst **Subgroup I d** and **Subgroup I e**

p_4_: p value for comparison amongst **Subgroup I d** and **Subgroup I f**

p_5_: p value for comparison amongst **Subgroup I e** and **Subgroup I f**

*: Statistical significance at p ≤ 0.05

#: Significant with Infected untreated (I b)@: Significant with Blank ZnO NPs (I c)

♦: Significant with Albendazole (I d)♣: Significant with NTZ (I e) and NTZ-loaded ZnO NPs (If)

**Table 2:** Counts of *T. spiralis* larvae in the muscles and drug efficacy in the various subgroups.

	Subgroup II b (Infected untreated control) (n = 6)	Subgroup II c (Blank ZnO NPs) (n = 6)	Subgroup II d (Albendazole) (n = 6)	Subgroup II e (NTZ) (n = 6)	Subgroup II f (NTZ-loaded ZnO NPs) (n = 6)
***T. spiralis* larval count in muscles**					
Min.	58300	36200	16800	13770	1700
Max.	65000	38000	17300	14330	1910
Mean	61310	37300^#^	17080^#@^	14076^#@♦^	1772^#@♦♣^
±SD.	2684.9	626.1	172.1	189.4	77.3
** F (p)**	2133.866^*^ (<0.001^*^)				
**p**_**1**_		<0.001^*^	<0.001^*^	<0.001^*^	<0.001^*^
**p**_**2**_			<0.001^*^	<0.001^*^	<0.001^*^
**Sig. bet. grps.**			p_3_ = 0.003^*^, p_4_ < 0.001^*^, p_5_ < 0.001^*^		
**Drug efficacy**		**39.16**	**72.14**	**77.04**	**97.11**

SD: **Standard deviation**

**Drug efficacy:** according to infected untreated control

**F: F for One way ANOVA test. Post Hoc Test (Tukey)** was done for pairwise comparison amongst each 2 subgroups

p: p value for comparison between the different studied groups

p_1_: p value for comparison amongst **Subgroup II b** and each other subgroup

p_2_: p value for comparison amongst **Subgroup II c** and each other subgroup

p_3_: p value for comparison amongst **Subgroup II d** and **Subgroup II e**

p_4_: p value for comparison amongst **Subgroup II d** and **Subgroup II f**

p_5_: p value for comparison amongst **Subgroup II e** and **Subgroup II f**

*: Statistical significance at p ≤ 0.05

#: Significant with Infected untreated (II b)

@: Significant with Blank ZnO NPs (II c)

♦: Significant with Albendazole (II d)

♣: Significant with NTZ (II e)

#### 2.2. Biochemical assessment.

Several serum biochemical markers (CK, ALT, AST and ALP) were analyzed in the present work. CK is primarily a marker of muscle damage which rises when *T. spiralis* larvae infiltrate the muscle tissues. ALT and AST are liver enzymes released into the bloodstream when liver cells are damaged. Trichinellosis, being a systemic infection, can lead to liver injury, either directly during larvae migration or through systemic inflammation. ALP is an enzyme related to liver function and its elevation indicates liver damage or a general systemic inflammatory response. All these serum biochemical markers were analyzed to evaluate the degree of the muscle or the liver injury induced by the infection and to assess the efficacy of treatment in alleviating this damage [23].

**Fig 5:**
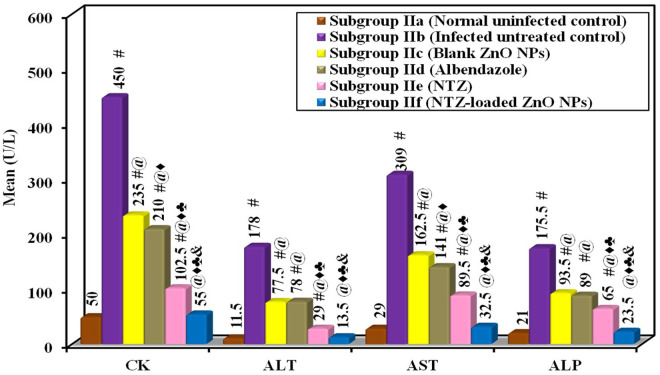
Biochemical changes in the sera of various subgroups with statistical comparison.

In the present study, all the biochemical serum markers (CK, ALT, AST and ALP) showed a marked significant increase in their levels in the infected untreated control compared to the normal uninfected mice. All treatments revealed a statistically significant reduction in the levels of all biochemical serum markers compared to the infected untreated control. Almost normalized results were observed among mice treated with NTZ-loaded ZnO NPs without a statistically significant difference in comparison with the normal uninfected control (Fig 5 and Table 3).

**Table 3:** Biochemical changes in the sera of various subgroups with statistical comparison.

	Subgroup II a (Normal uninfected control) (n = 6)	Subgroup II b (Infected untreated control) (n = 6)	Subgroup II c (Blank ZnO NPs) (n = 6)	Subgroup II d (Albendazole) (n = 6)	Subgroup II e (NTZ) (n = 6)	Subgroup II f (NTZ-loaded ZnO NPs) (n = 6)
**CK (U/L)**						
Min.	40	440	230	200	100	50
Max.	60	460	240	220	105	60
Mean	50	450^#^	235^#@^	210^#@♦^	102.5^#@♦♣^	55^@♦♣&^
±SD.	10.28	8.22	4.82	9.12	2.43	4.86
** F (p)**	2685.078^*^ (<0.001^*^)					
**p**_**1**_		<0.001^*^	<0.001^*^	<0.001^*^	<0.001^*^	0.830
**p**_**2**_			<0.001^*^	<0.001^*^	<0.001^*^	<0.001^*^
**p**_**3**_				<0.001^*^	<0.001^*^	<0.001^*^
**Sig. bet. grps.**				p_4_ < 0.001^*^, p_5_ < 0.001^*^, p_6_ < 0.001^*^		
**ALT (U/L)**						
Min.	11	176	75	76	28	13
Max.	12	180	80	80	30	14
Mean	11.50	178^#^	77.50^#@^	78^#@^	29^#@♦♣^	13.50^@♦♣&^
±SD.	0.55	1.90	2.43	1.90	0.89	0.55
F (p)	9883.221^*^ (<0.001^*^)					
p_1_		<0.001^*^	<0.001^*^	<0.001^*^	<0.001^*^	0.255
p_2_			<0.001^*^	<0.001^*^	<0.001^*^	<0.001^*^
p_3_				0.993	<0.001^*^	<0.001^*^
Sig. bet. grps.				p_4_ < 0.001^*^, p_5_ < 0.001^*^, p_6_ < 0.001^*^		
AST (U/L)						
Min.	28	308	150	132	89	32
Max.	30	310	175	150	90	33
Mean	29	309^#^	162.5^#@^	141^#@♦^	89.50^#@♦♣^	32.50^@♦♣&^
±SD.	0.89	0.89	11.26	7.85	0.55	0.55
** F (p)**	2060.613^*^ (<0.001^*^)					
**p**_**1**_		<0.001^*^	<0.001^*^	<0.001^*^	<0.001^*^	0.887
**p**_**2**_			<0.001^*^	<0.001^*^	<0.001^*^	<0.001^*^
**p**_**3**_				<0.001^*^	<0.001^*^	<0.001^*^
**Sig. bet. grps.**				p_4_ < 0.001^*^, p_5_ < 0.001^*^, p_6_ < 0.001^*^		
**ALP (U/L)**						
Min.	20	171	92	88	60	22
Max.	22	180	95	90	70	25
Mean	21	175.5^#^	93.50^#@^	89^#@^	65^#@♦♣^	23.5^@♦♣&^
±SD.	0.89	3.89	1.38	0.89	4.56	1.38
** F (p)**	2833.925^*^ (<0.001^*^)					
**p**_**1**_		<0.001^*^	<0.001^*^	<0.001^*^	<0.001^*^	0.573
**p**_**2**_			<0.001^*^	<0.001^*^	<0.001^*^	<0.001^*^
**p**_**3**_				0.059	<0.001^*^	<0.001^*^
**Sig. bet. grps.**				p_4_ < 0.001^*^, p_5_ < 0.001^*^, p_6_ < 0.001^*^		

SD: **Standard deviation**

**F: F for One way ANOVA test. Post Hoc Test (Tukey)** was done for pairwise comparison amongst each 2 subgroups

p: p value for comparison amongst the different subgroups

p_1_: p value for comparison amongst **Subgroup II a** and each other subgroup

p_2_: p value for comparison amongst **Subgroup II b** and each other subgroup

p_3_: p value for comparison amongst **Subgroup II c** and each other subgroup

p_4_: p value for comparison amongst **Subgroup II d** and **Subgroup II e**

p_5_: p value for comparison amongst **Subgroup II d** and **Subgroup II f**

p_6_: p value for comparison amongst **Subgroup II e** and **Subgroup II f**

*: Statistical significance at p ≤ 0.05

#: Significant with Normal uninfected (II a)

@: Significant with Infected untreated (II b)

♦: Significant with Blank ZnO NPs (II c)

♣: Significant with Albendazole (II d)

&: Significant with NTZ (II e) and NTZ-loaded ZnO NPs (II f)

*T. spiralis* infection induces an inflammatory response that generates free radicals, leading to oxidative tissue damage. Thus, oxidative stress tissue markers (MDA and NO) were analyzed in the present study. MDA is a byproduct of lipid peroxidation that serves as a key marker for oxidative stress. NO has a dual role in infection; it is part of the immune response but it can additionally contribute to tissue damage through reactive nitrogen species formation. Thus, raised NO levels indicate inflammation and oxidative stress. Measuring MDA and NO levels provides insight into the extent of oxidative damage during trichinellosis and whether the treatment dampens this response [24].

**Fig 6:**
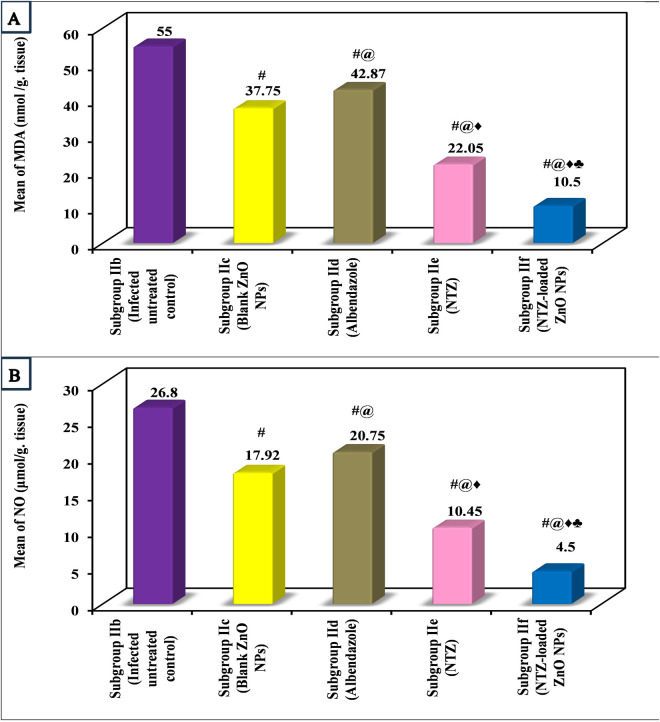
Biochemical changes in the muscle tissues of various subgroups. (A) Mean MDA (nmol/g tissue) among various subgroups. (B) Mean NO (μmol/g tissue) among various subgroups.

Results of the oxidative stress tissue markers (MDA and NO) in the current work also demonstrated the highest mean result in infected untreated control. All the treated subgroups revealed a remarkable decrease in the oxidative markers. Remarkably, NTZ-loaded ZnO NPs showed the lowest levels, followed by NTZ, ZnO NPs and finally albendazole (Fig 6 and Table 4).

**Table 4:** Biochemical changes in the muscle tissues of various subgroups with statistical comparison.

	Subgroup II b (Infected untreated control) (n = 6)	Subgroup II c (Blank ZnO NPs) (n = 6)	Subgroup II d (Albendazole) (n = 6)	Subgroup II e (NTZ) (n = 6)	Subgroup II f (NTZ-loaded ZnO NPs) (n = 6)
**MDA** **(nmol/g. tissue)**					
Min.	53.0	37.50	42.70	22.0	10.0
Max.	57.0	38.0	43.0	22.10	11.0
Mean	55.0	37.75^#^	42.87^#@^	22.05^#@♦^	10.50^#@♦♣^
±SD.	1.67	0.21	0.12	0.05	0.55
** F (p)**	2914.418^*^ (<0.001^*^)				
**p**_**1**_		<0.001^*^	<0.001^*^	<0.001^*^	<0.001^*^
**p**_**2**_			<0.001^*^	<0.001^*^	<0.001^*^
**Sig. bet. grps.**			p_3_ < 0.001^*^, p_4_ < 0.001^*^, p_5_ < 0.001^*^		
**NO (μmol/g. tissue)**					
Min.	25.70	17.80	20.50	10.0	4.0
Max.	28.0	18.0	21.0	10.90	5.0
Mean	26.80	17.92^#^	20.75^#@^	10.45^#@♦^	4.50^#@♦♣^
±SD.	0.93	0.09	0.22	0.39	0.45
** F (p)**	1812.675^*^ (<0.001^*^)				
**p**_**1**_		<0.001^*^	<0.001^*^	<0.001^*^	<0.001^*^
**p**_**2**_			<0.001^*^	<0.001^*^	<0.001^*^
**Sig. bet. grps.**			p_3_ < 0.001^*^, p_4_ < 0.001^*^, p_5_ < 0.001^*^		

SD: **Standard deviation**

**F: F for One way ANOVA test. Post Hoc Test (Tukey)** was done for pairwise comparison amongst each 2 subgroups

p: p value for comparison amongst the different subgroups

p_1_: p value for comparison amongst **Subgroup II b** and each other subgroup

p_2_: p value for comparison amongst **Subgroup II c** and each other subgroup

p_3_: p value for comparison amongst **Subgroup II d** and **Subgroup II e**

p_4_: p value for comparison amongst **Subgroup II d** and **Subgroup II f**

p_5_: p value for comparison amongst **Subgroup II e** and **Subgroup II e**

*: Statistical significance at p ≤ 0.05

#: Significant with Infected untreated (II b)

@: Significant with Blank ZnO NPs (II c)

♦: Significant with Albendazole (II d)

♣: Significant NTZ (II e) and NTZ-loaded ZnO NPs (II f)

Relying on the observed reductions in the levels of all the studied biochemical markers (CK, ALT, AST, ALP, MDA and NO), NTZ-loaded ZnO NPs demonstrated a protective effect against both the muscle and liver damage, as well as the oxidative stress induced by trichinellosis. This suggests a better control over infection-related damage compared to the other used treatments.

#### 2.3. Immunological assessment.

The immune response to *T. spiralis* infection includes Th1 response (IL-2 and IFN-γ) and type 2 cytokine response (IL-4, IL-5, IL-9, IL-10 and IL-13) [25,26]. IL-2 was analyzed because it exhibits apparent suppressive effect on the infectivity of *T. spiralis* [27]. IL-4 was chosen as it exerts an essential role in both the protective and pathological responses to *T. spiralis* infection [28].

**Fig 7:**
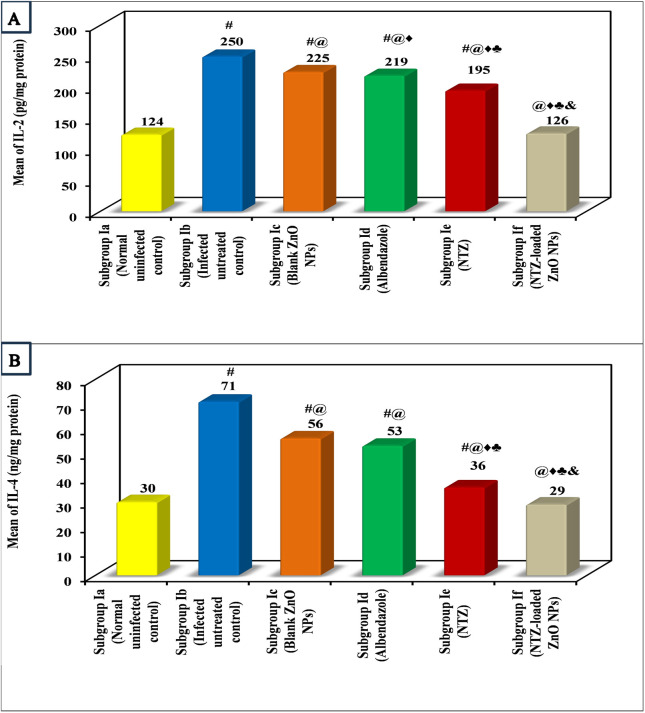
Immunological changes in the intestinal phase among various subgroups. (A) Mean IL-2 (pg/mg protein) among various subgroups. (B) Mean IL-4 (ng/mg protein) among various subgroups.

**Fig 8:**
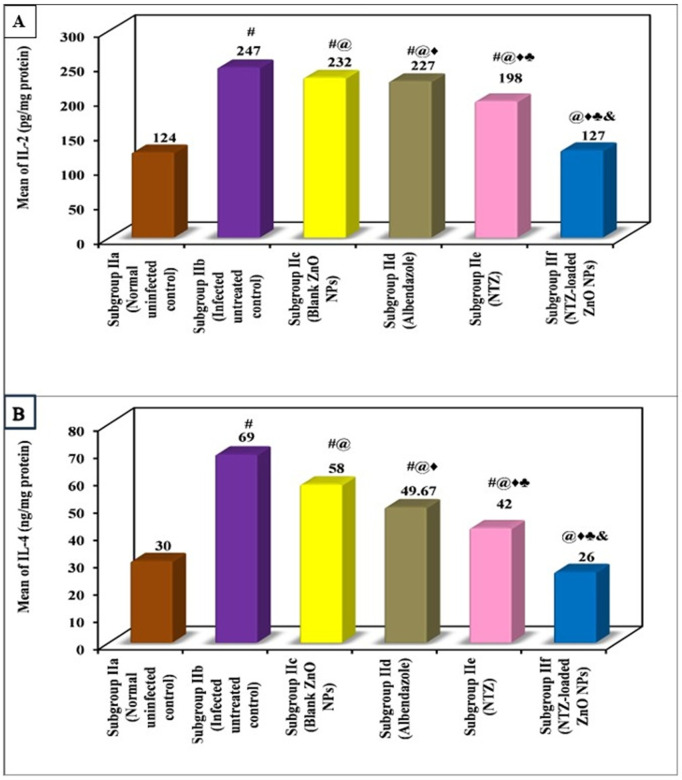
Immunological changes in the muscular phase among various subgroups. (A) Mean IL-2 (pg/mg protein) among various subgroups. (B) Mean IL-4 (ng/mg protein) among various subgroups.

Immunological results of the present study showed that the infection induced a highly significant rise in the levels of both IL-2 and IL-4 compared to normal uninfected control in both the intestinal and muscular phases. All lines of treatment were capable of decreasing the levels of IL-2 and IL-4 when compared to infected untreated control in the intestinal phase as well as the muscular one. Nonetheless, treatment with NTZ-loaded ZnO NPs almost normalized the levels of both ILs in either phase with no significant difference in comparison with normal uninfected control. NTZ came in the second place followed by albendazole with a significant difference between them (Figs 7 and 8, and Tables 5 and 6).

**Table 5:** Immunological intestinal changes of various subgroups with statistical comparison.

	Subgroup I a (Normal uninfected control) (n = 6)	Subgroup I b (Infected untreated control) (n = 6)	Subgroup I c (Blank ZnO NPs) (n = 6)	Subgroup I d (Albendazole) (n = 6)	Subgroup I e (NTZ) (n = 6)	Subgroup I f (NTZ-loaded ZnO NPs) (n = 6)
**IL-2** **(pg/mg protein)**						
Min.	122.0	248.0	223.0	217.0	193.0	124.0
Max.	126.0	252.0	227.0	221.0	197.0	128.0
Mean	124.0	250.0^#^	225.0^#@^	219.0^#@♦^	195.0^#@♦♣^	126.0^@♦♣&^
±SD.	1.79	1.79	1.79	1.79	1.79	1.79
** F (p)**	5303.563*(<0.001*)					
**p**_**1**_		<0.001^*^	<0.001^*^	<0.001^*^	<0.001^*^	0.401
**p**_**2**_			<0.001^*^	<0.001^*^	<0.001^*^	<0.001^*^
**p**_**3**_				<0.001^*^	<0.001^*^	<0.001^*^
**Sig. bet. grps.**				p_4_ < 0.001^*^, p_5_ < 0.001^*^, p_6_ < 0.001^*^		
**IL-4** **(ng/mg protein)**						
Min.	28.0	69.0	54.0	51.0	34.0	27.0
Max.	32.0	73.0	58.0	55.0	38.0	31.0
Mean	30.0	71.0^#^	56.0^#@^	53.0^#@^	36.0^#@♦♣^	29.0^@♦♣&^
±SD.	1.79	1.79	1.79	1.79	1.79	1.79
** F (p)**	532.063^*^(<0.001^*^)					
**p**_**1**_		<0.001^*^	<0.001^*^	<0.001^*^	<0.001^*^	0.924
**p**_**2**_			<0.001^*^	<0.001^*^	<0.001^*^	<0.001^*^
**p**_**3**_				0.068	<0.001^*^	<0.001^*^
**Sig. bet. grps.**				p_4_ < 0.001^*^, p_5_ < 0.001^*^, p_6_ < 0.001^*^		

SD: **Standard deviation**

**F: F for One way ANOVA test. Post Hoc Test (Tukey)** was done for pairwise comparison amongst each 2 subgroups

p: p value for comparison amongst the different subgroups

p_1_: p value for comparison amongst Subgroup I a and each other subgroup

p_2_: p value for comparison amongst **Subgroup I b** and each other subgroup

p_3_: p value for comparison amongst **Subgroup I c** and each other subgroup

p_4_: p value for comparison amongst **Subgroup I d** and **Subgroup I e**

p_5_: p value for comparison amongst **Subgroup I d** and **Subgroup I f**

p_6_: p value for comparison amongst **Subgroup I e** and **Subgroup I f**

*: Statistical significance at p ≤ 0.05

#: Significant with Normal uninfected (I a)

@: Significant with Infected untreated control (I b)

♦: Significant with Blank ZnO NPs (I c)

♣: Significant with albendazole (I d)

&: Significant with NTZ (I e) and NTZ-loaded ZnO NPs (I f)

**Table 6:** Immunological muscular changes of various subgroups with statistical comparison.

	Subgroup II a (Normal uninfected control) (n = 6)	Subgroup II b (Infected untreated control) (n = 6)	Subgroup II c (Blank ZnO NPs) (n = 6)	Subgroup II d (Albendazole) (n = 6)	Subgroup II e (NTZ) (n = 6)	Subgroup II f (NTZ-loaded ZnO NPs) (n = 6)
**IL-2** **(pg/mg protein)**						
Min.	122.0	245.0	230.0	225.0	196.0	125.0
Max.	126.0	249.0	234.0	229.0	200.0	129.0
Mean	124.0	247.0^#^	232.0^#@^	227.0^#@♦^	198.0^#@♦♣^	127.0^@♦♣&^
±SD.	1.79	1.79	1.79	1.79	1.79	1.79
** F (p)**	5525.063^*^(<0.001^*^)					
**p**_**1**_		<0.001^*^	<0.001^*^	<0.001^*^	<0.001^*^	0.068
**p**_**2**_			<0.001^*^	<0.001^*^	<0.001^*^	<0.001^*^
**p**_**3**_				<0.001^*^	<0.001^*^	<0.001^*^
**Sig. bet. grps.**				p_4_ < 0.001^*^, p_5_ < 0.001^*^, p_6_ < 0.001^*^		
**IL-4** **(ng/mg protein)**						
Min.	28.0	67.0	56.0	46.0	40.0	24.0
Max.	32.0	71.0	60.0	55.0	45.0	28.0
Mean	30.0	69.0^#^	58.0^#@^	49.67^#@♦^	42.0^#@♦♣^	26.0^@♦♣&^
±SD.	1.79	1.79	1.79	4.23	2.37	1.79
** F (p)**	269.632^*^(<0.001^*^)					
**p**_**1**_		<0.001^*^	<0.001^*^	<0.001^*^	<0.001^*^	0.082
**p**_**2**_			<0.001^*^	<0.001^*^	<0.001^*^	<0.001^*^
**p**_**3**_				<0.001^*^	<0.001^*^	<0.001^*^
**Sig. bet. grps.**				p_4_ < 0.001^*^, p_5_ < 0.001^*^, p_6_ < 0.001^*^		

SD: **Standard deviation**

**F: F for One way ANOVA test. Post Hoc Test (Tukey)** was done for pairwise comparison amongst each 2 subgroups

p: p value for comparison amongst the different subgroups

p_1_: p value for comparison amongst **Subgroup II a** and each other subgroup

p_2_: p value for comparison amongst **Subgroup II b** and each other subgroup

p_3_: p value for comparison amongst **Subgroup II c** and each other subgroup

p_4_: p value for comparison amongst **Subgroup II d** and **Subgroup II e**

p_5_: p value for comparison amongst **Subgroup II d** and **Subgroup II f**

p_6_: p value for comparison amongst **Subgroup II e** and **Subgroup II f**

*: Statistical significance at p ≤ 0.05

#: Significant with Normal uninfected (II a)

@: Significant with Infected untreated control (II b)

♦: Significant with Blank ZnO NPs (II c)

♣: Significant with Albendazole (II d)

&: Significant with NTZ (II e) and NTZ-loaded ZnO NPs (II f)

Normalization of both ILs by NTZ-loaded ZnO NPs in both phases indicates that the formula was effective in the suspension of the immune response against trichinellosis.

#### 2.4. Histopathological assessment.

**Fig 9:**
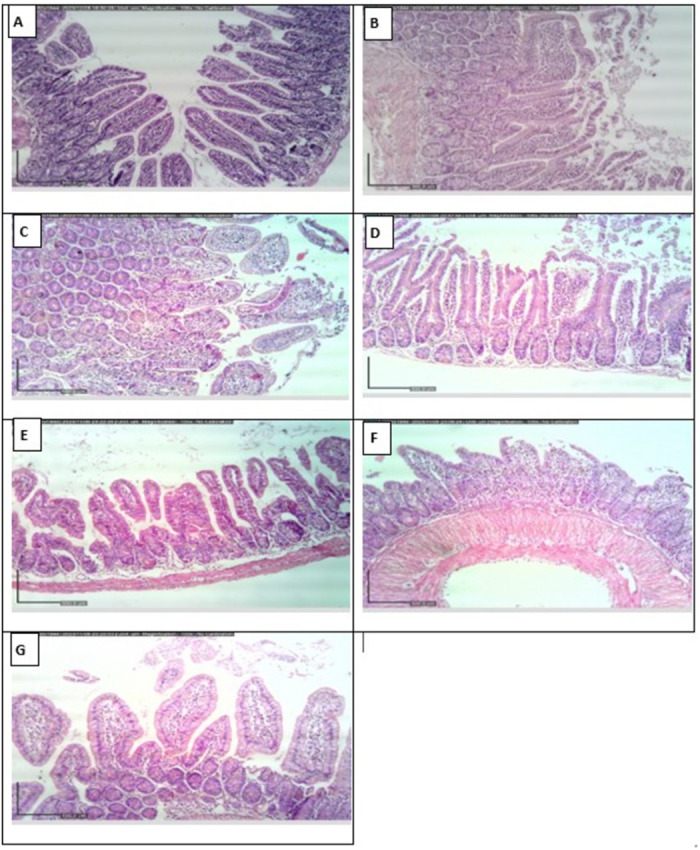
Intestinal histopathologic photomicrographs (H&E). (A) Intestinal mucosa of normal uninfected control representing normal villous architecture with minimal chronic inflammatory cell infiltrate (x100). (B) and (C) Intestine of infected untreated control shows moderate to marked mixed inflammatory infiltrate, villous broadening with focal fusion (x100). (D) Blank ZnO NPs treated mice reveals moderate villous broadening with infiltration by moderate mixed inflammation (x100). (E) Albendazole treated subgroup displays intestine with mild inflammation and focal villous fusion (x100). (F) Mice treated with NTZ demonstrate focal villous broadening with mild mixed inflammation (x100). (G) Well-formed villi with minimal inflammation are noticed in mice treated with NTZ-loaded ZnO NPs (x100).

***2.4.1. Histopathological alterations in intestine***. The intestine of mice from infected untreated control subgroup revealed viable adult worms. Moderate to marked mixed inflammatory infiltrate were observed accompanied with villi broadening, focal fusion and blunting. Histopathologic assessment of the different treated subgroups revealed a reduction in the intestinal inflammatory response as well as less villous affection. However, an almost normalized histologic improvement was noticed in the subgroup treated with NTZ-loaded ZnO NPs with statistically significant difference when compared to infected untreated control (Fig 9 and Table 7).

**Table 7:** Histopathological intestinal changes of various subgroups with statistical comparison.

	Subgroup I b (Infected untreated control) (n = 6)	Subgroup I c (Blank ZnO NPs) (n = 6)	Subgroup I d (Albendazole) (n = 6)	Subgroup I e (NTZ) (n = 6)	Subgroup I f (NTZ-loaded ZnO NPs) (n = 6)
Intestinal changes				^#^	^#@♣^
Normal	0 (0%)	0 (0%)	1 (16.7%)	0(0%)	5 (83.3%)
Mild	0 (0%)	0 (0%)	3 (50%)	4 (66.7%)	1 (16.7%)
Moderate	2 (33.3%)	5 (83.3%)	2 (33.3%)	2 (33.3%)	0 (0%)
Marked	4 (66.7%)	1 (16.7%)	0 (0%)	0(0%)	0 (0%)
χ2 (p)	28.359*(<0.001*)				
p_1_		^FE^p = 0.242	^MC^p = 0.054	^MC^p = 0.034*	^MC^p = 0.003*
p_2_			^MC^p = 0.114	^MC^p = 0.056	^MC^p = 0.003*
Sig. bet. grps.			^MC^p_3_ = 1.000, ^MC^p_4_ = 0.111, ^MC^p_5_ = 0.028^*^		

χ^2^: **Chi square test**MC: **Monte Carlo**FE: **Fisher Exact**

p: p value for comparison amongst the different studied groups

p_1_: p value for comparison amongst **Subgroup I b** and each other subgroup

p_2_: p value for comparison amongst **Subgroup I c** and each other subgroup

p_3_: p value for comparison amongst **Subgroup I d** and **Subgroup I e**

p_4_: p value for comparison amongst **Subgroup I d** and **Subgroup I f**

p_5_: p value for comparison amongst Subgroup I e and **Subgroup I f**

*: Statistical significance at p ≤ 0.05

#: **Subgroup I b** (Infected untreated control

@: **Subgroup I c** (Blank ZnO NPs)

♣: Significant NTZ (I e)

**Fig 10:**
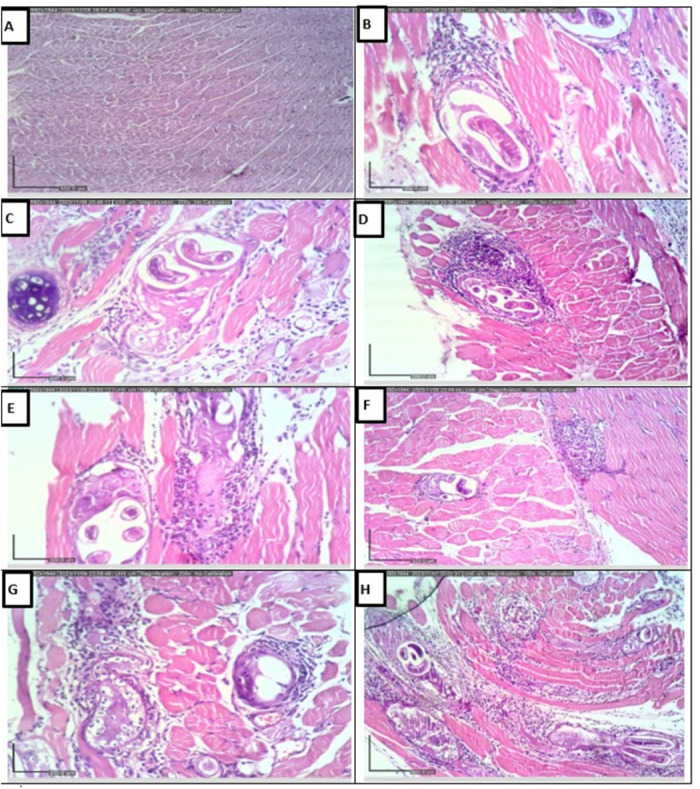
Muscular histopathologic photomicrographs (H&E). (A) Skeletal muscle tissue of normal uninfected control representing normal architecture (x100). (B) Muscle of infected untreated control reveals encysted larva with surrounding thick capsule and mild inflammatory reaction (x200). (C) Blank ZnO NPs treated mice displays viable larva surrounded by thick muscular capsule with mild inflammatory reaction (x200). (D) Mice treated with albendazole show larva surrounded by a thin capsule with moderate mixed inflammation (x100). (E) and (F) NTZ treated subgroup demonstrates some degenerated larvae with surrounding disrupted capsules attacked by moderate to marked inflammation (x200 and x100 respectively). (G) and (H) NTZ-loaded ZnO NPs with the mostly degenerated larvae, evidently disrupted capsules that are strongly attacked by marked mixed inflammatory infiltrate (x200 and x100 respectively).

***2.4.2. Histopathological alterations in muscle.*** A thick capsule with mild inflammatory reaction was noticed among the muscle sections of the infected untreated control. Among all the treated subgroups, larvae degeneration along with thinning or disruption in the muscular capsules were observed. Additionally, changes in the pericapsular inflammatory infiltrate were notably observed among the different treated subgroups. All these variations were remarkably detected in the mice treated with NTZ-loaded ZnO NPs. The latter indicated the most larval degeneration accompanied with capsular disruption and an evident attack by a mixed inflammatory infiltrate. All these changes among this subgroup represented statistically significant difference when compared to infected untreated control (Fig 10 and Table 8).

**Table 8:** Histopathological muscular changes of various subgroups with statistical comparison.

	Subgroup II b (Infected untreated control) (n = 6)	Subgroup II c (Blank ZnO NPs) (n = 6)	Subgroup II d (Albendazole) (n = 6)	Subgroup II e (NTZ) (n = 6)	Subgroup II f (NTZ-loaded ZnO NPs) (n = 6)
Muscle capsule					^#^
Thick	6 (100%)	5 (83.3%)	4 (66.7%)	3 (50%)	1 (16.7%)
Thin/disrupted	0 (0.0%)	1 (16.7%)	2 (33.3%)	3 (50%)	5 (83.3%)
χ2 (p)	9.989*(0.037*)				
^FE^p_1_		1.000	0.455	0.182	0.015*
^FE^p_2_			1.000	0.545	0.080
Sig. bet. grps.			^FE^p_3_ = 1.000, p_4_ = 0.242, p_5_ = 0.545		
Muscle inflammation					^#@^
Mild	4 (66.7%)	3 (50%)	1 (16.7%)	0 (0%)	0 (0%)
Moderate	2 (33.3%)	3 (50%)	4 (66.7%)	5 (83.3%)	2 (33.3%)
Marked	0 (0%)	0 (0%)	1 (16.7%)	1 (16.7%)	4 (66.7%)
χ2 (p)	14.816*(0.022*)				
^MC^p_1_		1.000	0.244	0.059	0.039*
^MC^p_2_			0.539	0.187	0.039*
Sig. bet. grps.			^MC^p_3_ = 1.000, p_4_ = 0.238, p_5_ = 0.242		

χ^2^: **Chi square test**MC: **Monte Carlo**FE: **Fisher Exact**

p: p value for comparison amongst the different studied subgroups

p_1_: p value for comparison amongst **Subgroup I b** and each other subgroup

p_2_: p value for comparison amongst **Subgroup I c** and each other subgroup

p_3_: p value for comparison amongst **Subgroup I d** and Subgroup I e

p_4_: p value for comparison amongst **Subgroup I d** and **Subgroup I f**

p_5_: p value for comparison amongst Subgroup I e and **Subgroup I f**

* : Statistical significance at p ≤ 0.05

#: Subgroup II b (Infected untreated control)

@: Subgroup II c (Blank ZnO NPs)

## Discussion

Although trichinellosis is a serious disease which exhibits a worldwide prevalence, its treatment options are still scarce. The most commercially used albendazole has multiple fatal adverse effects such as epilepsy and encephalitis. Additionally, it expresses a reduced efficacy against the encysted larval stage. Consequently, exploring an alternative anthelminthic therapy with safety and effectiveness on the intestinal and muscular phases of *T. spiralis* is a chief target in medicinal research [29]. NTZ is a commonly used therapy for various intestinal helminths as well as protozoa offering a wide tolerance level. Nevertheless, its low solubility is an obstacle which contributes to cure rates of 80% in giardiasis and 36.6% in acute fascioliasis when utilized as a substitute for the failure of triclabendazole. This in turn, necessitates the development of the currently available drugs to benefit from their enormous privileges [1,8].

An innovative frontier in drug delivery systems is presented by the nanotechnology science in order to defeat the limitations of the commercially available drugs [16,30]. NPs with the size of up to hundreds of nanometers have the power to penetrate across the tissues. Consequently, this would augment the permeability and reduce the dose of the utilized drugs. Furthermore, nanotechnology offers a cost-effective strategy as the development of a nano-vehicle for the conventional drugs is much cheaper than discovering new therapeutic regimens [8]. ZnO nano-vehicles have gained considerable reputation over other metal oxides due to their biocompatible safe anti-microbial effect that is approved by the FDA, along with their potent capability of raising the solubility of the loaded therapeutic agents [19–22].

The main aim of this study was to prepare a nanometric-sized formulation of NTZ loaded on ZnO NPs with the intention of enhancing the biological action and tissue penetrability of the utilized drug. The nano-formula was used for the first time in the treatment of the intestinal and muscular phases of experimental trichinellosis.

The results of the NPs characterization denoted that both the blank ZnO NPs and NTZ-loaded ZnO NPs possess spherical and hexagonal structures with a mean size of 30.37 and 80.87 nm respectively. This increased size indicates the successful loading of NTZ on the ZnO NPs. The smaller particles size offers a higher surface area to volume ratio which in turn leads to easier penetration across the cell membranes. Besides, small-sized NPs owe a lower in vivo toxicity with better tissue distribution [31].

FTIR spectroscopy exhibited absorption peaks of blank ZnO NPs at 598.88 and 609.88 cm-1 compared to 464.04, 501.74, 533.39 and 563.83 cm-1 of NTZ-loaded ZnO NPs. These peaks correspond to metal-oxygen (ZnO stretching) vibration mode while the shift in the peaks indicates successful NTZ loading on ZnO NPs [32].

The diffraction peaks in the XRD pattern ensured that both formulations have grown in polycrystalline nature with spherical and hexagonal crystal structures. This was illustrated in the appearance of a number of XRD peaks attributing to different crystalline orientations. Hence, this indicates the effective NTZ loading on ZnO NPs. Additionally, the sharp diffraction peaks of the synthesized formulae indicate their good crystallinity [33].

NTZ-loaded ZnO NPs exhibited an EE of 66% denoting the drug loading. This is usually desirable as it boosts the likelihood to transport and release sufficient drug amount across the body tissues [7].

Evaluation of parasite count highpoints the severity of infection and possesses the capability of assessing the treatment effectiveness [8,34]. Throwing light on the utilized treatments during the intestinal phase, all the used regimens produced a significant reduction in the adults count compared to infected untreated control. ZnO NPs presented a decrease in parasite count with a significant difference in comparison with infected untreated control. This can be justified by the known anti-helminthic action of ZnO that damages the pathogen membrane through electrostatic binding. The formed membrane disruptions ease the NPs penetration leading to profounder lesions [35,36]. Both albendazole and NTZ showed an evident reduction in the parasite burden with no significant difference between them. The anthelmintic effect of both regimens against the intestinal adult worms was proven in previous studies [1,7,29]. Albendazole mechanism of action was explained by the selective interaction with the parasite tubulin. This in turn, leads to discontinuing the microtubule formation needed in cell division. Even though, resistance may occur as a result of the formation of an altered tubulin by the parasites, which reduces the chance of binding to albendazole [7,37]. On the other hand, the mechanism of NTZ was recognized by inhibiting the pyruvate ferredoxin oxidoreductase enzyme that disturbs parasite respiratory metabolism, in addition to causing surface membrane lesions [8]. Despite of the generally safe nature of NTZ, it still owes a reduced efficacy as a result of its reduced solubility [1,8]. Curiously, NTZ-loaded ZnO NPs revealed the highest significant drug efficacy exceeding 97%. Loading of NTZ on ZnO NPs supplemented a prodigious benefit via improving drug solubility that consequently enhanced the drug tissue permeability and efficacy. Additionally, the prepared nano-formula presented an anti-parasitic synergistic effect of both ZnO and NTZ. This synchronizes with previous researches that documented the effectiveness of the nano-metric formulae in decreasing parasite counts compared to free drugs [7,8].

The parasite count during the muscular phase almost followed the same pattern as in the intestinal phase. All the treatments attained a significant decrease in larval count in comparison with infected untreated control. Unlikely, NTZ demonstrated a better efficacy compared to albendazole with a significant difference between them. This can be clearly explained by the known reduced effect of albendazole on the muscular encysted larvae [1,7]. Similar to the intestinal phase, NTZ-loaded ZnO NPs highlighted the highest significant drug efficacy in muscles which exceeded 97%.

The levels of the studied serum biochemical markers (CK, ALT, AST and ALP) displayed a significant rise in infected untreated control compared to treated subgroups, indicating the impact of the infection on these markers. CK elevation can be attributed to skeletal muscle injury while ALT, AST and ALP elevations denote liver damage induced by larval migration [6,38]. Previous research has corroborated with these findings which highlighted the association between infection and elevated markers levels [38]. Nonetheless, treatment demonstrated a notable reduction in these markers across all treated subgroups. This suggests the efficacy of the interventions in ameliorating the biochemical perturbations induced by *T. spiralis* infection [38]. On the same line, the current study’s findings revealed that NTZ-loaded ZnO NPs successfully improved the biochemical parameters to be in the normal range.

The over production of oxidative markers in response to parasitic infections is considered as a major cause of subsequent tissue damage [39]. Consequently, the oxidative stress markers of MDA and NO were studied in the current work. Both oxidative markers demonstrated the highest mean levels in the infected untreated control, scoring the uppermost oxidative stress associated with the infection. This observation aligns with existing literature documenting the relationship between infection and increased oxidative stress [38]. Interestingly, all treated subgroups exhibited a reduction in oxidative markers, with NTZ-loaded ZnO subgroup showing the most pronounced decrease. These findings are consistent with studies that focused on the prominent effect of NTZ as well as ZnO NPs in mitigating oxidative stress [1,40]. This striking reduction in oxidative markers further supports the potential therapeutic benefit of the used nano-formula in alleviating oxidative stress associated with infection.

Results of the immunological assessment denoted a significant rise of IL-2 and IL-4 in the intestinal as well as the muscular phase of the infected untreated control. Remarkably, NTZ-loaded ZnO NPs was the only exclusive subgroup that nearly normalized the levels of both ILs of the two phases with no significant difference compared to the normal uninfected control. This can be elucidated by the potential value of ZnO in controlling the oxidative stress and regulating the inflammatory cytokines [40–42]. All the results coincide with other studies that indicated the rise of inflammatory ILs in *T. spiralis* infected mice along with their restoration in successfully treated groups [38,43]. The immunological pattern during *T. spiralis* infection was explained by previous studies which illustrated that the immune system is activated by different parasite molecules, present on the adult’s cuticle or in the excretory-secretory products [44]. To overcome the parasites invasion, the host’s innate and adaptive immune responses are stimulated and commence the process of expulsion. During the infection, the cellular immune response presents a mixed Th1/Th2 immune response with Th2 predominance during the chronic stage [45]. Interaction of helminth-derived molecules with the host immune system can initiate a shift from inflammatory to anti-inflammatory immune response. Throughout the intestinal phase, the immune response is mixed against *T. spiralis*; Th1 response, designated by raised interferon gamma (IFN-γ), IL-2, and IL-12 that are predominant at the initial stage, and the Th2 response, characterized by elevated IL-4, IL-5, IL-9, IL-10, and IL-13 [44,45]. However, with establishment of the immunomodulatory action of *T. spiralis* in the muscular phase, some cytokine production increase as IL-4, IL-5, IL-9, IL-10, IL-13, IL-21, and IL-33 and other cytokines decrease as IFN γ [25,46–51].

The present study confirmed the achieved results through the histopathological study of the intestine and muscle. Regarding the histopathological results during the intestinal phase, infected untreated control revealed inflammatory infiltration along with broadening and blunting of villi. All the used treatments ameliorated all these destructive changes. Meanwhile, the most statistically significant improvement was observed among the mice treated with NTZ-loaded ZnO NPs compared to infected untreated control. Other studies reported similar findings of infected untreated control, albendazole and NTZ- treated mice [1,7,52].

Histopathologic examination of the muscle biopsies of infected untreated control demonstrated larvae surrounded by thick capsule and inflammatory reaction. The treated subgroups illustrated the degeneration of larvae in thinned or disrupted capsules accompanied with pericapsular inflammatory infiltration. Attentively, these changes were mostly noticed in the mice treated with NTZ-loaded ZnO NPs which represented the only significant subgroup when compared to infected untreated control. This can be explained by the marked inflammatory attack which contributed to the foremost larvae degeneration and capsules disruption [7,51]. The immune response and gene expression in the host and parasite metabolism are switched on at the different developmental phases. Neutrophils are vital elements during host innate immune responses and play vital roles in local immunity during infection by promoting inflammation and conquering infections. Neutrophils can regulate adaptive immune responses by inhibiting or stimulating T cell proliferation, according to activation status [53–55].

## Materials and methods

### 1. Ethics statement

The experimental work of the current study was evaluated and accepted by the Ethics Committee of the Medical Research Institute, Alexandria University, Egypt complying with ICLAS (International Council for Laboratory Animal Science) guidelines (protocol approval serial number: 012239332).

### 2. Parasite and experimental animals

*T. spiralis* strain was obtained from the Parasitology Department, Theodor Bilharz Research Institute (TBRI), Giza, Egypt. Seventy-two Swiss Albino male mice, each of 20-25 grams, have been used. The infection dose was adjusted to 250 orally inoculated *T. spiralis* larvae/mouse. All mice were kept in plastic cages having full accessibility to standard pelleted food and water under optimal housing conditions (12 hours light and 12 hours dark cycles at 25-27 °C). [6,7,56,57]

### 3. Drugs

The drugs administered in the present study included:

- Albendazole: The commercial Alzental oral suspension from Epico pharmaceutical company, Egypt.
- NTZ: The commercial Nanazoxid oral suspension from Utopia pharmaceutical company, Egypt.
- Blank ZnO NPs: Zinc acetate dihydrate from Alpha Chemika, India. Sodium hydroxide (NaOH) from Techno Pharmachem, India. HPLC-grade dimethyl sulfoxide (DMSO) and acetone were acquired from Tedia, USA. Deionized water was used for the solution preparation.
- NTZ-loaded ZnO NPs (NTZ pure powder was generously supplied by Utopia pharmaceuticals, Egypt).

#### 3.1. Preparation of blank ZnO NPs.

ZnO NPs were prepared through using direct precipitation technique relying on two precursors; zinc acetate dihydrate and NaOH. The aqueous solutions of both zinc acetate dehydrate (0.1 M) as well as sodium hydroxide (0.2 M) were prepared. The NaOH solution was added drop-wisely to a beaker containing the zinc acetate dehydrate solution with continuous stirring at 600 rpm for two hours at room temperature. The formed white precipitate was separated through subsequent centrifugation at 10,000 rpm for 15 mins along with washing with deionized water for three times followed by acetone washing. The precipitate was finally oven-dried at 120 °C for 6 hours in order to reach the stable ZnO NPs [58,59].

#### 3.2. Preparation of NTZ-loaded ZnO NPs.

In order to prepare NTZ-loaded ZnO NPs, 30 mg of the prepared ZnO NPs were added to 10 ml of deionized water followed by sonication for 15 mins. Additionally, 30 mg of NTZ was dissolved in 10 ml of DMSO, followed by stirring at 600 rpm for 15 mins. The two solutions were then mixed using magnetic stirrer at 600 rpm. After six hours, NTZ-loaded ZnO NPs were separated by subsequent centrifugation at 10,000 rpm for 15 mins along with washing with deionized water for three times. Finally, the formed precipitate was dried in oven below 80 °C [59].

#### 3.3. Characterization of blank ZnO NPs and NTZ-loaded ZnO NPs.

***3.3.1. Transmission electron microscopy (TEM)***. TEM was used to determine the shape and size of NPs. Samples were subjected to negative staining by 2% uranyl acetate, and investigated using TEM (JEOL-100 CX, Tokyo, Japan) at an acceleration voltage of 10 kV [16].

***3.3.2. Fourier transform infrared (FTIR) spectroscopy***. FTIR (Thermo Scientific, USA) was utilized to detect the functional groups of the prepared NPs. FTIR spectra of both blank ZnO NPs and NTZ-loaded ZnO NPs were analyzed in a wave number range of 4000 to 400 cm^-1^ [7].

***3.3.3. X-ray diffractometry (XRD)***. The XRD (Bruker D8 discover, Coventry, UK) was used to analyze the crystallinity nature of the prepared nano-formulations. It relied on Cu-Kα radiation source operating at λ of 1.54060 Å, 10 mA and 30 kV with an angle adjustment in a range of 2θ = 5˚to 80˚ and scan rate of 0.0505˚. The diffraction patterns of the analyzed NPs were then compared with the Joint Committee on Powder Diffraction Standards (JCPDS) (card number: 36-1451) [60].

***3.3.4. Entrapment efﬁciency (EE)***. The EE of the amount of NTZ in NTZ-loaded ZnO NPs was calculated by the indirect method. Briefly, 2 ml aliquot of the prepared NTZ-loaded ZnO NPs was ultra-centrifuged at 10,000 rpm for 15 min at 4°C using a cooling centrifuge (Beckman Optima TM, Indianapolis, IN). The proportion of the free unloaded NTZ in the supernatant was spectrophotometrically analyzed (Shimadzu, model UV-1800 PC, Kyoto, Japan) against a blank at 340 nm. Then, the EE % was estimated using the equation [8,61]:

$\begin{array}{c} EE\%=\frac{Total\;concentration\;of\;NTZ\;-Concentration\;of\;free\;NTZ\;in\;supernatant}{Total\;concentration\;of\;NTZ}\;\times \;100. \end{array}$

### 4. Experimental design

The study included 72 mice. Except for the normal uninfected control subgroups, each mouse was orally inoculated with 250 *T. spiralis* larvae [7,56].

Mice were further allocated into two main equal groups (36 mice/group)

- Group I: Intestinal phase (36 mice)
  - Subgroup I a (6 mice): Normal uninfected control so that each mouse daily received 100 μl oral saline (the vehicle of drugs suspension) by gavage syringe [7].
  - Subgroup I b (6 mice): *T. spiralis* infected untreated control so that each mouse daily received 100 μl oral saline [7].
  - Subgroup I c (6 mice): Infected mice were orally treated with blank ZnO NPs in daily dose of 10 mg/kg for three days [62].
  - Subgroup I d (6 mice): Infected mice were orally treated with albendazole in daily dose of 50 mg/kg for three days [7].
  - Subgroup I e (6 mice): Infected mice were orally treated with NTZ in daily dose of 50 mg/kg for three days [1].
  - Subgroup I f (6 mice): Infected mice were orally treated with NTZ-loaded ZnO NPs in daily dose of 50 mg/kg for three days.

Treatments in this group were given on the 2^nd^ day post-infection (P.I) and mice were sacrificed on the 6^th^ day P.I to assess the therapeutic effectiveness on the intestinal phase [7].

Each intestinal specimen was divided into two parts. A small part was fixed in formalin for the histopathological study while the other part was used for the parasitological evaluation of the adult count [7].

- Group II: Muscular phase (36 mice)
  - Subgroup II a (6 mice): Normal uninfected control so that each mouse daily received 100 μl oral saline (the vehicle of drugs suspension) by gavage syringe [7].
  - Subgroup II b (6 mice): *T. spiralis* infected untreated control so that each mouse daily received 100 μl oral saline [7].
  - Subgroup II c (6 mice): Infected mice were orally treated with blank ZnO NPs in daily dose of 10 mg/kg for three days [62].
  - Subgroup II d (6 mice): Infected mice were orally treated with albendazole in daily dose of 50 mg/kg for three days [7].
  - Subgroup II e (6 mice): Infected mice were orally treated with NTZ in daily dose of 50 mg/kg for three days [1].
  - Subgroup II f (6 mice): Infected mice were orally treated with NTZ-loaded ZnO NPs in daily dose of 50 mg/kg for three days.

Treatments in this group were given on day 30 P.I and mice were sacrificed on day 35 P.I following the treatment completion in order to assess the therapeutic efficacy on the muscular phase [7].

Muscle samples attained from one hind leg were stored at - 20˚C for further tissue biochemical assessment while the muscles obtained from the other hind leg were preserved in 10% formalin for the histopathological study. The rest of mice muscles were digested for the parasitological evaluation of the larval count [1,7].

At the end of either the intestinal or muscular phases, 0.5 ml blood was collected from the retro-orbital blood vessels of each mouse into clean Eppendorf tube, left to coagulate for 20 mins at room temperature followed by centrifugation at 3000 rpm for 10 mins. The serum was separated, divided into aliquots and stored at - 80˚C until used for further biochemical and immunological analysis [63].

### 5. Evaluation of the therapeutic efficacy

#### 5.1. Parasitological assessment.

***5.1.1. Counting T. spiralis adults in the intestine***. In order to evaluate the treatment efficacy on the intestinal phase, infected mice of group I were sacrificed and the intestine of each mouse was longitudinally opened and washed. The intestine was then cut into pieces with 1 cm each, added in 10 ml saline, incubated for 2 hours at 37 °C in order to allow the adult migration out of the intestinal tissues. The saline in the container was collected in tubes and the intestine was furtherly washed several times. All the collected fluid was centrifuged for 5 mins at 1500 rpm. The supernatant was discarded, follwed by the reconstitution of sediment in drops of saline. The adult worms were microscopically counted at 10 x magnification [1,7,63].

***5.1.2. Counting T. spiralis larvae in the muscles***. In order to assess the treatment efficacy on the muscular phase, infected mice of group II were sacrificed, skinned and eviscerated. Muscles were digested in 1% pepsin-concentrated hydrochloric acid. The mixture was then incubated for 2 hours at 37 °C with continous stirring using a magnetic stirrer. Sieving of the digested product was performed. Collection of larvae was achieved via repeated sedimentation technique along with washing three times with saline where the supernatant was decanted each time. The larvae in the sediment were microscopically counted using a McMaster counting chamber at 10 x magnification [1,29,64–66].

***5.1.3. Drug efficacy***. Efficacy (%) of each drug was estimated using the following equation [7,67]:

$Efficacy=\;\frac{\begin{array}{c} Adults\;or\;larvae\;count\;in\;infected\;untreated\;control\;subgroup\\ -Adults\;or\;larvae\;count\;in\;treated\;subgroup\; \end{array}}{Adults\;or\;larvae\;count\;in\;infected\;untreated\;control\;subgroup}\times 100\backslash )$

#### 5.2. Biochemical assessment.

***5.2.1. Serum biochemical assessment***. Serum biochemical markers were analyzed in the samples that were collected at the end of the muscular phase. Beckman Coulter AU480 automated clinical chemistry analyzer was used for high-throughput testing of various biochemical parameters. Serum creatine kinase (CK) enzyme was evaluated as a biomarker of muscle involvement. Serum alanine aminotransferase (ALT), aspartate aminotransferase (AST) and alkaline phosphatase (ALP) were assessed as indicators of liver toxicity [38].

***5.2.2. Tissue biochemical assessment***. Muscle tissues that were stored at -20 °C were homogenized in cold buffered solution (0.5 g Na_2_HPO_4_ and 0.7 g NaH_2_PO_4_ dissolved in 500 ml deionized water at pH of 7.4). Centrifugation was performed at 4°C for 15 mins at 4000 rpm then the supernatant was isolated for further biochemical analysis. Oxidative stress tissue markers (malondialdehyde (MDA) and nitric oxide (NO)) were measured in the muscle homogenates by enzyme-linked immunosorbent assay (ELISA) method using commercially available kit (Bio-diagnostic Company, Egypt) [68].

#### 5.3. Immunological assessment.

Interleukin (IL)-2 and IL4 levels were assessed in serum samples of both the intestinal and the muscular phases by ELISA technique using commercially available kits (Elabscience, USA and Quantikine, USA) respectively [68].

#### 5.4. Histopathological assessment.

Tissue samples from small intestines as well as hind leg skeletal muscles of all studied subgroups were fixed in 10% neutral buffered formalin for 24 hours. Dehydration was performed in ascending grades of alcohol, cleared in xylene and then embedded into paraffin blocks. Sections were cut, each of four microns thick and stained with haematoxylin and eosin (H&E). Slides were examined microscopically to evaluate the histopathologic changes. All the histopathological samples were randomized followed by coding and blind examination [7].

Scoring of intestinal changes was evaluated in terms of intestinal inflammatory infiltrate along with villi changes in the form of broadening, fusion and blunting (normal, mild, moderate or marked) [7].

Scoring of muscular changes was assessed in view of thickness of cysts capsules (thick or thin/focally disrupted) and pericapsular inflammatory infiltrates (mild, moderate or marked) [7].

### 6. Statistical analysis of the data

Data analysis was performed by utilizing IBM SPSS software package, version 20.0 (Armonk, NY: IBM Corp). Quantitative data were represented as range (minimum and maximum), mean and standard deviation. For normally distributed quantitative variables, One Way ANOVA test was used for comparing the studied subgroups and followed by Post Hoc test (Tukey) for pairwise comparison. Categorical data were expressed as numbers and percentages. Chi-square test was used in order to compare between two subgroups. Alternatively, Fisher Exact or Monte Carlo correction tests were applied when more than 20% of the cells had expected count less than 5. The significance of all attained results was adjudicated at 5% level [69].

## Conclusion

NTZ-loaded ZnO NPs offered a promising vista in treating both the intestinal and muscular phases of murine trichinellosis regarding the studied parasitological, biochemical, immunological and histopathological parameters. In order to reach an auspicious treatment platform, further toxicological studies and testing the full cytokine profile are recommended to prevail over the limitations.
